# Enhancing disease surveillance with novel data streams: challenges and opportunities

**DOI:** 10.1140/epjds/s13688-015-0054-0

**Published:** 2015-10-16

**Authors:** Benjamin M Althouse, Samuel V Scarpino, Lauren Ancel Meyers, John W Ayers, Marisa Bargsten, Joan Baumbach, John S Brownstein, Lauren Castro, Hannah Clapham, Derek AT Cummings, Sara Del Valle, Stephen Eubank, Geoffrey Fairchild, Lyn Finelli, Nicholas Generous, Dylan George, David R Harper, Laurent Hébert-Dufresne, Michael A Johansson, Kevin Konty, Marc Lipsitch, Gabriel Milinovich, Joseph D Miller, Elaine O Nsoesie, Donald R Olson, Michael Paul, Philip M Polgreen, Reid Priedhorsky, Jonathan M Read, Isabel Rodríguez-Barraquer, Derek J Smith, Christian Stefansen, David L Swerdlow, Deborah Thompson, Alessandro Vespignani, Amy Wesolowski

**Affiliations:** 1 Santa Fe Institute, Santa Fe, NM USA; 2 The University of Texas at Austin, Austin, TX USA; 3 San Diego State University, San Diego, CA USA; 4 New Mexico Department of Health, Santa Fe, NM USA; 5 Children’s Hospital Informatics Program, Boston Children’s Hospital, Boston, MA USA; 6 Department of Pediatrics, Harvard Medical School, Boston, MA USA; 7 Department of Epidemiology, Biostatistics and Occupational Health, McGill University, Montreal, QC Canada; 8 Defense Systems and Analysis Division, Los Alamos National Laboratory, Los Alamos, NM USA; 9 Department of Epidemiology, Johns Hopkins Bloomberg School of Public Health, Baltimore, MD USA; 10 Virginia BioInformatics Institute and Department of Population Health Sciences, Virginia Tech, Blacksburg, VA USA; 11 Influenza Division, Centers for Disease Control and Prevention, Atlanta, GA USA; 12 Biomedical Advanced Research and Development Authority (BARDA), Assistant Secretary for Preparedness and Response (ASPR), Department of Health and Human Services, Washington, DC USA; 13 Chatham House, 10 St James’s Square, London, SW1Y 4LE UK; 14 Division of Vector-Borne Diseases, NCEZID, Centers for Disease Control and Prevention, San Juan, PR USA; 15 Division of Epidemiology, New York City Department of Health and Mental Hygiene, New York, NY USA; 16 Communicable Disease Dynamics, Harvard School of Public Health, Boston, MA USA; 17 School of Population Health, The University of Queensland, Brisbane, QLD Australia; 18 Division of Vector-Borne Diseases, NCEZID, Centers for Disease Control and Prevention, Atlanta, GA USA; 19 Department of Computer Science, Johns Hopkins University, Baltimore, MD USA; 20 University of Iowa, Iowa City, IA USA; 21 Department of Epidemiology and Population Health, Institute of Infection and Global Health, University of Liverpool, Liverpool, CH64 7TE UK; 22 Health Protection Research Unit in Emerging and Zoonotic Infections, NIHR, Liverpool, L69 7BE UK; 23 Department of Zoology, University of Cambridge, Cambridge, CB2 3EJ UK; 24 Google Inc., Mountain View, CA USA; 25 National Center for Immunization and Respiratory Diseases, Centers for Disease Control and Prevention, Atlanta, GA USA; 26 Laboratory for the Modeling of Biological and Socio-technical Systems, Northeastern University, Boston, MA USA

**Keywords:** disease surveillance, novel data streams, digital surveillance

## Abstract

Novel data streams (NDS), such as web search data or social media updates, hold promise for enhancing the capabilities of public health surveillance. In this paper, we outline a conceptual framework for integrating NDS into current public health surveillance. Our approach focuses on two key questions: What are the opportunities for using NDS and what are the minimal tests of validity and utility that must be applied when using NDS? Identifying these opportunities will necessitate the involvement of public health authorities and an appreciation of the diversity of objectives and scales across agencies at different levels (local, state, national, international). We present the case that clearly articulating surveillance objectives and systematically evaluating NDS and comparing the performance of NDS to existing surveillance data and alternative NDS data is critical and has not sufficiently been addressed in many applications of NDS currently in the literature.

**Electronic Supplementary Material:**

The online version of this article (doi:10.1140/epjds/s13688-015-0054-0) contains supplementary material.

## What are novel data streams?

We define NDS as those data streams whose content is initiated directly by the user (patient) themselves. This would exclude data sources such as electronic health records, disease registries, vital statistics, electronic lab reporting, emergency department visits, ambulance call data, school absenteeism, prescription pharmacy sales, serology, amongst others. Although ready access to aggregated information from these excluded sources is novel in many health settings, our focus here is on those streams which are both directly initiated by the user and also not already maintained by public health departments or other health professionals. Despite this more narrow definition our suggestions for improving NDS surveillance may also be applicable to more established surveillance systems, participatory systems (e.g., Flu Near You, influenzaNet) [1, 2], and new data streams aggregated from established systems, such as Biosense 2.0 and ISDS DiSTRIBuTE network [3, 4].

While much of the recent focus on using NDS for disease surveillance has centered on Internet search queries [5, 6] and Twitter posts [7, 8], there are many NDS outside of these two sources. Our aim therefore is to provide a general framework for enhancing and developing NDS surveillance systems, which applies to more than just search data and Tweets. At a minimum, our definition of NDS would include Internet search data and social media, such as Google searches, Google Plus, Facebook, and Twitter posts, as well as Wikipedia access logs [9, 10], restaurant reservation and review logs [11, 12], non-prescription pharmacy sales [13, 14], news source scraping [15], and prediction markets [16].

## How does NDS integrate into the surveillance ecosystem?

**Figure 1:**
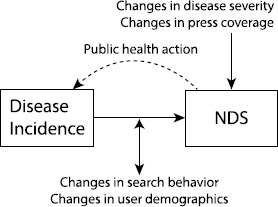
**The link between public health problems and NDS is modified by user behavior (i.e., propensity to search, what terms are chosen to search, etc.), user demographics, external forces on user behavior (i.e., changing disease severity, changing press coverage, etc.), and finally by public health interventions, which by design aim to modify the public health problem creating feedback loops on the link to NDS.**

Using NDS for surveillance or in supporting public health decision making necessitates an understanding of the complex link between the time-varying public health problems (i.e., disease incidence) and the time-varying NDS signal. As illustrated in Figure 1, this link is modified by user behavior (i.e., propensity to search, what terms are chosen to search, etc.), user demographics, external forces on user behavior (i.e., changing disease severity, changing press coverage, etc.), and finally by public health interventions, which by design aim to modify the public health problem creating feedback loops on the link to NDS. As a result, developing NDS-based surveillance systems presents a number of challenges, many of which are comparable to those faced by systems comprised of more established data sources such as physician visits or laboratory test results.

NDS could add value to existing surveillance in several ways. NDS can increase the timeliness of surveillance information, improve temporal or spatial resolution of surveillance, add surveillance to places with no existing systems, improve dissemination of data, measure unanticipated outcomes of interest (i.e. a syndrome associated with a new pathogen that is not currently under surveillance in an established system), measure aspects of a transmission/disease process not captured by traditional surveillance (i.e. behavior, perception), and increase the population size under surveillance.

The most studied example of the potential benefits and unique challenges associated with NDS comes from Google Flu Trends. In 2008, Google developed an algorithm which translates search queries into an estimate of the number of individuals with influenza-like illness that visit primary healthcare providers [17]. The original goal of Google Flu Trends (GFT) was to provide accessible data on influenza-like illness in order to reduce reporting delays, increase the spatial resolution of data, and provide information on countries outside the United States of America [17]. GFT has added value to existing surveillance for influenza. However, although there has been some benefit both to academic researchers and public health practitioners, GFT has also received criticism [18, 19].

Much of the recent criticism of GFT seems to stem from two issues: the first is the effect of changing user behavior during anomalous events [19, 20] and the second is whether real-time, nowcasting of influenza using GFT adds value to the existing systems available to public health authorities. The first criticism, changing behavior during anomalous events, is an issue for both existing systems and proposed systems based on NDS. The key difference is that existing systems may be both better understood and easier to validate in real-time. While such criticisms may not undermine the case for use of NDS, they do emphasize that the validation of any NDS approach is an ongoing process, and even a perfectly validated system in one period or location may become uncalibrated as behaviors change. It is therefore not meaningful to say that a particular NDS system is or is not informative; that statement must be qualified in space and time. Moreover, the fact that decalibration to “gold standard” systems cannot be detected immediately but only in retrospect is another reason why NDS can only supplement and never fully replace such systems. The second criticism, the need for nowcasting, may depend on the user’s access to different data sources. For public health authorities with access to high-resolution data on reported cases of influenza, simple autoregressive models can be used to nowcast with high accuracy [19]. However, access to these high resolution data-sets varies by public health level (local, state, federal, and international) as well as by user group: researchers, public health authorities, and the private sector. As a result, the utility of GFT varies by user, but for those without access to high-resolution data, it remains an important source of information.

Since the release of GFT, similar NDS-based systems have been developed to extend surveillance to places where resource or other constraints limit the availability of direct clinical or laboratory surveillance data and improve the timeliness of detection and forecasting of disease incidence. For example, NDS have facilitated expansion of dengue and influenza surveillance to countries without infrastructure capable of real time surveillance [5, 17, 21, 22]. This has also been done in the context of hospitalizations in Texas [23], mental illness, psychological manifestations of physical morbidities [24, 25], and search queries from clinical decision support sites, such as UpToDate [26]. In these cases, although NDS-based systems are being asked to estimate data that is actually being collected, those data are not available quickly enough for use in public health decision making.

As stated earlier, in some cases NDS can be used to assess behavior - something that remains a challenge for traditional case-based surveillance. Although this is a challenge for translating NDS signals into estimates of disease incidence, it presents a unique opportunity to study health seeking behavior. For example, NDS has facilitated an exploration of population-level changes in health-related behaviors following changes in tobacco related policy [27, 28] or after unpredictable events such as celebrity deaths or cancer diagnoses [29, 30]. NDS can help us understand and monitor health-related behavior, but little recent work has focused on this area. How does vaccination sentiment respond to changes in disease prevalence? How is health-seeking behavior discussed in social networks? Does that information dissemination manifest in action? Answering these questions accurately may require integration of Twitter, Facebook, Wikipedia access logs, web searches or web search logs, hospitalization records, and EMR with existing measures of behavior such as the Behavioral Risk Factor Surveillance System. As a result, it is critically important to understand the user’s intent; for example, what are the behavioral, biological, and/or epidemiological underpinnings of information-seeking online? A Google or Wikipedia search for the keyword “ulcer”, for instance, is likely a response to having symptoms of an ulcer while a search for “h pylori” is more likely a response to something more specific, such as a lab confirmed test for an ulcer-causative agent. Similarly, posting a Tweet about a “healthy recipe” is likely a different action than searching for a “healthy recipe”; where the former is an act of broadcasting information, while the latter is an act of searching for information. This suggests that large-scale experiments combining NDS could explore these behaviors.

Therefore, in order to address the challenges associated with NDS-based surveillance and properly integrate NDS into existing systems, we advocate for a three-step system: (1) Quantitatively define the surveillance objective(s); (2) build the surveillance systems and model(s) by adding data (existing and novel) in until there is no additional improvement in model performance to achieve stated objectives, assessed by (3) performing rigorous validation and testing. These steps are comparable to those prescribed for evaluating more established systems [31].

## How do we ensure the robustness of NDS surveillance systems?

NDS, by their very definition, do not have a long track record of use. As a result, rigorous standards for validating NDS and systems constructed using NDS must be adopted. These validation procedures should include both best practices in machine learning and also best practices from surveillance system design such as the proportion of persons identified that are true positives for the disease under surveillance [31]. Building on previous work [10], we have systematically evaluated the existing published NDS surveillance papers using the following criteria: was validation used and if so what type, are the data open and if not why not, and is the code open source. While we understand that it’s not always possible, due to privacy concerns and data use agreements, to make data open access, it’s essential that the community be able to externally validate methods and NDS. Therefore, a component of validation must be the use of data that is publicly available (or at least available to researchers) for training and testing of NDS. Of 66 papers identified, only 27 (41%) performed any validation, only one [5] stated that the source code was available, and while some used publicly available data, no papers publicly shared the data used in their analyses. (see Table 1 and Table S1 in Additional file 1).

**Table 1:** **The use of open source code and validation across papers using NDS for surveillance**

	**Validation**	**No validation**
Open source code	1/66 (1.50%)	0/66 (0%)
No open source code	26/66 (39.4%)	39/66 (59.1%)

While the lack of validation is troubling, there is a deeper issue: it may be the case that many existing standards for validation are inadequate for use on disease surveillance systems using NDS [32, 33]. For example, there are well-documented cases of failure when the training set does not contain important dynamics of the system [34–36]. For that reason we advocate for model development by repeated training and testing on subsets of the data and that a final, validation set be held back entirely during model construction [35]. This final validation set should be used only once, at the conclusion of the study. Ideally, this final set will be completely blind to the developer. Lastly, after these development and validation steps, models should be openly evaluated prospectively to further support their validity. Put simply, this approach could be summarized as internal validation, external validation, and continued prospective evaluation. While these steps help to ensure the validity of models, it may be that given the volatile nature of disease processes and human behavior (non-linear and non-stationary dynamics), it may be technically impossible to design robust surveillance systems using proxy data and regression models alone.

Validation must also be conducted by other researchers. First, transparency of methods and reproducibility of forecasts is essential to both the scientific process and in examining the utility of models/NDS. Second, new methods or NDS must demonstrate improvements upon existing methods or data sources. Performance can be over-stated by comparing performance of NDS systems with trivial instances of traditional models. Clear definition of appropriate baseline models and their definition is critical to assessing the improvement of new models utilizing novel streams. Without open access to data and code, these crucial steps are not possible. Ideally, manuscripts would report, in detail, the methods employed, provide open-source code implementing those methods, and make the data used to generate the prediction available. Despite legitimate concerns about privacy, data use agreements between agencies, and the often substantial effort required to gather data, we must work towards ensuring our scientific publications are replicable and useful for evaluating the next generation of surveillance tools.

Validation can also be conducted by complementary studies. For example, researchers could conduct studies on how users interact with NDS sources, such as Google or Twitter. These detailed studies would provide valuable information on potential biases and suggest mechanisms for improving the robustness of surveillance systems constructed from these NDS. The need for these focused studies again highlights the utility of collaboration of private sector companies, such as Google and Twitter, with researchers and public health practitioners. Recent efforts by Google and Twitter to better engage with the research community represents an important first step.

## What is the future of NDS surveillance?

NDS should provide robust, long-term surveillance solutions. Even after EMR are at the fingertips of public health decision-makers and researchers, NDS will provide a snapshot of activity, which is unrelated to the medical encounter. Therefore, a critical first step when evaluating NDS for surveillance is determining what problems are likely to be short term and what problems are likely to be longer term. Again, clearly defined quantitative surveillance goals must be the most important components of NDS-driven systems.

A second important distinction is that surveillance needs, potential benefits, and general utility vary by country, region, and locality. For example, many state and local health agencies in the U.S. already have access to high-resolution, near real-time data for infectious diseases. In this case, local utility may be limited to understanding behavioral responses. These data are also useful, however, for validating these systems more generally. In regions where less data is available, the utility of models may be high but comprehensive evaluation may not be possible. Finally, both Internet and website (or app) penetration vary by geographic region.

Clearly, NDS cannot replace physician and laboratory data, though it can be used to augment the surveillance coming out of systems collecting that type of data. Furthermore, the need for model validation highlights the often-overlooked importance of maintaining traditional/existing systems in the existing NDS literature. Without these systems, it would be impossible to validate and update NDS-enabled systems.

As a community of researchers and public health decision makers, we must decide on how to proceed. Specifically, we must ensure stability and robustness of these NDS-based systems. Pure research is important, but if our goal is to design systems to support public health decisions, they must achieve a higher level of stability. Peer review of systems must carefully evaluate validation relative to established surveillance systems. This of course gives rise to the open question of who should be responsible for funding and maintaining these new systems. The future success of these efforts hinges on building and maintaining collaborations between private-sector, public health agencies, and academics. Finally, while the field has been critical of Google and GFT, it is because we are able to criticize: No other NDS-based system had continuously provided public health predictions for as long as GFT, many NDS surveillance systems had not been as carefully evaluated [6, 18–20], and fewer still had been implemented prospectively. Despite the recent cessation of GFT, Google provided a living system for NDS surveillance. Next generation surveillance systems using NDS hold great promise for improving the health of our global society. Realizing their potential will require more rigorous standards of validation and improved collaboration between researchers in academia, the private sector, and public health.

## Electronic Supplementary Material

Below is the link to the electronic supplementary material.

**Supplemental table** (pdf)
