# Supplementary material for: Enhancing disease surveillance with novel data streams: challenges and opportunities
Source: EPJ Data Sci. 2015 Oct 16;4(1):17. doi: 10.1140/epjds/s13688-015-0054-0 (PMC5156315; doi:10.1140/epjds/s13688-015-0054-0)
Supplement: Supplementary file 1 — Supplemental table (pdf) [file 13688_2015_54_MOESM1_ESM.pdf]

Supplemental Table for *Enhancing disease surveillance with novel data streams* by Althouse, Scarpino, *et al.*

Reference	System(s)	Open Source	Validation Method	Disease
Achrekar et al. [2011]	Twitter	No	5-fold cross validation	influenza
Achrekar et al. [2012]	Twitter	No	10-fold cross validation	influenza
Althouse et al. [2011]	Google	Yes	k-fold cross validation, leave one out, leave 1 year out, fit models forward and backward	dengue
Althouse et al. [2014]	Google	No	None	psychosomatic symptoms
Aramaki et al. [2012]	Google	No	None	influenza
Aramaki et al. [2011]	Twitter	No	None not clear	influenza
Ayers et al. [2011]	Google	No	None	smoking
Ayers et al. [2012]	Google	No	None	"psychological distress"
Ayers et al. [2013a]	Google	No	None	mental health
Ayers et al. [2013b]	Google	No	None	smoking
Ayers et al. [2014a]	Google	No	None	"healthy considerations"
Ayers et al. [2014b]	Google	No	None	cancer interest
Breyer et al. [2011]	Google	No	None	kidney stones
Broniatowski et al. [2013]	Twitter	No	10-fold cross-validation	influenza
Carneiro and Mylonakis [2009]	Google	No	None	west nile virus, RSV, avian influenza
Chan et al. [2011]	Google	No	Held out one season as testing set	dengue
Cho et al. [2013]	Google	No	None	influenza
Chunara et al. [2012]	Twitter	No	None	cholera
Cooper et al. [2005]	Yahoo	No	None	cancer
Corley et al. [2010]	Blogs	No	None	influenza
Culotta [2013]	Twitter	No	Fit and predict forward, leave one out	influenza
Desai et al. [2012]	Google	No	None	gastroenteritis
Doan et al. [2012]	Twitter	No	None	influenza
Dugas et al. [2013]	Google	No	Leave one season out	influenza
Dukic et al. [2011]	Google	No	None	MRSA

Continued on next page

Table 1 – *Continued from previous page*

Reference	System(s)	Open Source	Validation Method	Disease
Eysenbach [2006]	Google	No	None	influenza
Ginsberg et al. [2008]	Google	No	Held out testing sets, predicted forward	influenza
Gomide et al. [2011]	Twitter	No	None not clear	dengue
Hagihara et al. [2012]	Google	No	None	suicide
Hirose and Wang [2012]	Twitter	No	Training/test set	influenza
Hulth et al. [2010]	medical	No	None	gastroenteritis
Hulth et al. [2009]	medical	No	k-fold cross validation, leave one out	influenza
Hulth and Rydevik [2011]	medical	No	None	influenza
Ingram and Plante [2013]	Google	No	None	restless legs syndrome
Jia-xing et al. [2013]	Baidu	No	Predict forward	gonorrhea
Johnson et al. [2004]	medical	No	None	influenza
Kang et al. [2013]	Google	No	None	influenza
Kim et al. [2013]	Twitter	No	None not clear	influenza
Lamb et al. [2013]	Twitter	No	Mechanical turk validation of tweets	influenza
Lampos and Cristianini [2012]	Twitter	No	Separate training, validation, testing sets	influenza
Lampos and Cristianini [2010]	Twitter	No	Training/test set	influenza
Lindh et al. [2012]	Google	No	None	head lice
McIver and Brownstein [2014]	Wikipedia	No	Jackknife resampling, training/testing set	influenza
Nagel et al. [2013]	Twitter	No	None	influenza, pertussis
Noar et al. [2013]	Google	No	None	cancer interest
Nsoesie et al. [2014]	OpenTable	No	None	influenza
Ocampo et al. [2013]	Google	No	Training/test set, leave one year out	malaria
Paul and Dredze [2011]	Twitter	No	Training/test set	influenza
Pelat et al. [2009]	Google	No	None	influenza, gastroenteritis, chicken pox
Polgreen et al. [2008]	Yahoo	No	None	influenza, pneumonia
Santos and Matos [2013]	Twitter	No	Training/test set	influenza
Seifter et al. [2010]	Google	No	None	Lyme disease

Continued on next page

Table 1 – *Continued from previous page*

Reference	System(s)	Open Source	Validation Method	Disease
Scarpino et al. [2012]	Google	Yes	Training/test set	influenza
Shaman et al. [2013]	Google, Twitter	No	Retrospective forecasts	influenza
Signorini et al. [2011]	Twitter	No	Out of sample validation	influenza
Walcott et al. [2011]	Google	No	None	stroke
Wilson and Brownstein [2009]	Google	No	None	listeriosis
Won et al. [2013]	Blogs	No	Training/test set	suicide
Xu et al. [2010]	Google	No	Training/test set, cross validation	influenza
Xu et al. [2011]	Google, medical	No	Training/test set	HFMD
Yang et al. [2011]	Google	No	None	suicide
Yang et al. [2010]	Google	No	None	seasonal depression
Yuan et al. [2013]	Baidu	No	Training/test set	influenza
Zheluk et al. [2013]	Google, Yandex	No	None	HIV/AIDS
Zhou and Shen [2010]	Baidu	No	Training/test set	scarlet fever, dysentery, HIV/AIDS, tuberculosis
Zhou et al. [2011]	Google	No	Training/test set	influenza

References

H. Achrekar, A. Gandhe, R. Lazarus, Ssu-Hsin Yu, and Benyuan Liu. Predicting flu trends using Twitter data. In *Proc. Computer Communications Workshops (INFOCOM WKSHPS)*, pages 702–707. IEEE, 2011. doi: 10.1109/INFCOMW.2011.5928903.

Harshavardhan Achrekar, Avinash Gandhe, Ross Lazarus, Ssu-Hsin Yu, and Benyuan Liu. Twitter improves seasonal influenza prediction. In *Proc. Health Informatics (HEALTHINF)*, pages 61–70, 2012.

Benjamin M. Althouse, Yih Yng Ng, and Derek A. T. Cummings. Prediction of dengue incidence using search query surveillance. *PLOS Neglected Tropical Diseases*, 5(8):e1258, August 2011. doi: 10.1371/journal.pntd.0001258. URL `http://dx.doi.org/10.1371/journal.pntd.0001258`.

Benjamin M Althouse, Jon-Patrick Allem, Matthew A Childers, Mark Dredze, and John W Ayers. Population health concerns during the united states' great recession. *American journal of preventive medicine*, 46(2):166–170, 2014.

Eiji Aramaki, Sachiko Maskawa, and Mizuki Morita. Twitter catches the flu: Detecting influenza epidemics using Twitter. In *Proc. Empirical Methods in Natural Language Processing (EMNLP)*, pages 1568–1576. Association for Computational Linguistics, 2011. ISBN 978-1-937284-11-4. URL `http://dl.acm.org/citation.cfm?id=2145432.2145600`.

Eiji Aramaki, Sachiko Maskawa, and Mizuki Morita. Influenza patients are invisible in the Web: Traditional model still improves the state-of-the-art web based influenza surveillance. In *Proc. AAAI Spring Symposium*. AAAI, 2012. URL `http://www.aaai.org/ocs/index.php/SSS/SSS12/paper/download/4354/4669`.

John W. Ayers, Kurt Ribisl, and John S. Brownstein. Using search query surveillance to monitor tax avoidance and smoking cessation following the united states' 2009 "SCHIP" cigarette tax increase. *PLOS ONE*, 6(3):e16777, March 2011. doi: 10.1371/journal.pone.0016777. URL `http://dx.doi.org/10.1371/journal.pone.0016777`.

John W. Ayers, Benjamin M. Althouse, Jon-Patrick Allem, Matthew A. Childers, Waleed Zafar, Carl Latkin, Kurt M. Ribisl, and John S. Brownstein. Novel surveillance of psychological distress during the great recession. *Affective Disorders*, 142(1–3):323–330, December 2012. ISSN 0165-0327. doi: 10.1016/j.jad.2012.05.005. URL `http://www.sciencedirect.com/science/article/pii/S0165032712002893`.

John W. Ayers, Benjamin M. Althouse, Jon-Patrick Allem, J. Niels Rosenquist, and Daniel E. Ford. Seasonality in seeking mental health information on Google. *American Journal of Preventive Medicine*, 44(5):520–525, May 2013a. ISSN 0749-3797. doi: 10.1016/j.amepre.2013.01.012. URL `http://www.sciencedirect.com/science/article/pii/S0749379713000809`.

John W Ayers, Benjamin M Althouse, Kurt M Ribisl, and Sherry Emery. Digital detection for tobacco control: Online reactions to the united states' 2009 cigarette excise tax increase. *Nicotine & Tobacco Research*, page ntt186, 2013b.

John W Ayers, Benjamin M Althouse, Morgan Johnson, Mark Dredze, and Joanna E Cohen. What's the healthiest day?: Circaseptan (weekly) rhythms in healthy considerations. *American journal of preventive medicine*, 47(1):73–76, 2014a.

John W Ayers, Benjamin M Althouse, Seth M Noar, and Joanna E Cohen. Do celebrity cancer diagnoses promote primary cancer prevention? *Preventive medicine*, 58:81–84, 2014b.

Benjamin N. Breyer, Saunak Sen, David S. Aaronson, Marshall L. Stoller, Bradley A. Erickson, and Michael L. Eisenberg. Use of Google Insights for Search to track seasonal and geographic kidney stone incidence in the United States. *Urology*, 78(2):267–271, August 2011. ISSN 0090-4295. doi: 10.1016/j.urology.2011.01.010. URL `http://www.sciencedirect.com/science/article/pii/S0090429511000288`.

David A. Broniatowski, Michael J. Paul, and Mark Dredze. National and local influenza surveillance through Twitter: An analysis of the 2012–2013 influenza epidemic. *PLOS ONE*, 8(12):e83672, December 2013. doi: 10.1371/journal.pone.0083672. URL `http://dx.doi.org/10.1371/journal.pone.0083672`.

Herman Anthony Carneiro and Eleftherios Mylonakis. Google Trends: A Web-based tool for real-time surveillance of disease outbreaks. *Clinical Infectious Diseases*, 49(10):1557–1564, November 2009. ISSN 1058-4838, 1537-6591. doi: 10.1086/630200. URL `http://cid.oxfordjournals.org/content/49/10/1557`. PMID: 19845471.

Emily H. Chan, Vikram Sahai, Corrie Conrad, and John S. Brownstein. Using Web search query data to monitor dengue epidemics: A new model for neglected tropical disease surveillance. *PLOS Neglected Tropical Diseases*, 5(5):e1206, May 2011. doi: 10.1371/journal.pntd.0001206. URL `http://dx.doi.org/10.1371/journal.pntd.0001206`.

Sungjin Cho, Chang Hwan Sohn, Min Woo Jo, Soo-Yong Shin, Jae Ho Lee, Seoung Mok Ryoo, Won Young Kim, and Dong-Woo Seo. Correlation between national influenza surveillance data and Google Trends in South Korea. *PLOS ONE*, 8(12):e81422, December 2013. doi: 10.1371/journal.pone.0081422. URL http://dx.doi.org/10.1371/journal.pone.0081422.

Rumi Chunara, Jason R Andrews, and John S Brownstein. Social and news media enable estimation of epidemiological patterns early in the 2010 Haitian cholera outbreak. *American Journal of Tropical Medicine and Hygiene*, 86(1):39–45, January 2012. ISSN 0002-9637,. doi: 10.4269/ajtmh.2012.11-0597. URL http://www.ajtmh.org/content/86/1/39.

Crystale Purvis Cooper, Kenneth P Mallon, Steven Leadbetter, Lori A Pollack, and Lucy A Peipins. Cancer Internet search activity on a major search engine, United States 2001–2003. *Journal of Medical Internet Research*, 7(3), July 2005. ISSN 1438-8871. doi: 10.2196/jmir.7.3.e36. URL http://www.ncbi.nlm.nih.gov/pmc/articles/PMC1550657/. PMID: 15998627 PMCID: PMC1550657.

Courtney D. Corley, Diane J. Cook, Armin R. Mikler, and Karan P. Singh. Text and structural data mining of influenza mentions in Web and social media. *International Journal of Environmental Research and Public Health*, 7(2):596–615, February 2010. doi: 10.3390/ijerph7020596. URL http://www.mdpi.com/1660-4601/7/2/596.

Aron Culotta. Lightweight methods to estimate influenza rates and alcohol sales volume from Twitter messages. *Language Resources and Evaluation*, 47(1):217–238, March 2013. doi: 10.1007/s10579-012-9185-0.

Rishi Desai, Aron J. Hall, Benjamin A. Lopman, Yair Shimshoni, Marcus Rennick, Niv Efron, Yossi Matias, Manish M. Patel, and Umesh D. Parashar. Norovirus disease surveillance using Google Internet query share data. *Clinical Infectious Diseases*, 55(8):e75–e78, October 2012. ISSN 1058-4838, 1537-6591. doi: 10.1093/cid/cis579. URL http://cid.oxfordjournals.org/content/55/8/e75. PMID: 22715172.

S. Doan, L. Ohno-Machado, and N. Collier. Enhancing Twitter data analysis with simple semantic filtering: Example in tracking influenza-like illnesses. In *Proc. Healthcare Informatics, Imaging and Systems Biology (HISB)*, pages 62–71. IEEE, 2012. doi: 10.1109/HISB.2012.21.

Andrea Freyer Dugas, Mehdi Jalalpour, Yulia Gel, Scott Levin, Fred Torcaso, Takeru Igusa, and Richard E. Rothman. Influenza forecasting with Google Flu Trends. *PLOS ONE*, 8(2):e56176, February 2013. doi: 10.1371/journal.pone.0056176. URL http://dx.doi.org/10.1371/journal.pone.0056176.

Vanja M. Dukic, Michael Z. David, and Diane S. Lauderdale. Internet queries and methicillin-resistant Staphylococcus aureus surveillance. *Emerging Infectious Diseases*, 17(6):1068–1070, June 2011. ISSN 1080-6040, 1080-6059. doi: 10.3201/eid1706.101451.

Gunther Eysenbach. Infodemiology: Tracking flu-related searches on the Web for syndromic surveillance. *AMIA Annual Symposium*, 2006:244–248, 2006. ISSN 1942-597X. URL http://www.ncbi.nlm.nih.gov/pmc/articles/PMC1839505/. PMID: 17238340 PMCID: PMC1839505.

Jeremy Ginsberg, Matthew H. Mohebbi, Rajan S. Patel, Lynnette Brammer, Mark S. Smolinski, and Larry Brilliant. Detecting influenza epidemics using search engine query data. *Nature*, 457(7232), November 2008. ISSN 0028-0836, 1476-4687. doi: 10.1038/nature07634. URL http://www.nature.com/doifinder/10.1038/nature07634.

Janaína Gomide, Adriano Veloso, Wagner Meira Jr, Virgílio Almeida, Fabrício Benevenuto, Fernanda Ferraz, and Mauro Teixeira. Dengue surveillance based on a computational model of spatio-temporal locality of Twitter. In *Proc. Web Science Conference (WebSci)*, pages 1–8. ACM, 2011.

Akihito Hagihara, Shogo Miyazaki, and Takeru Abe. Internet suicide searches and the incidence of suicide in young people in Japan. *European Archives of Psychiatry and Clinical Neuroscience*, 262 (1):39–46, February 2012. ISSN 0940-1334, 1433-8491. doi: 10.1007/s00406-011-0212-8. URL http://link.springer.com/article/10.1007/s00406-011-0212-8.

H. Hirose and Liangliang Wang. Prediction of infectious disease spread using Twitter: A case of influenza. In *Proc. Parallel Architectures, Algorithms and Programming (PAAP)*, pages 100–105. IEEE, 2012. doi: 10.1109/PAAP.2012.23.

A Hulth and G Rydevik. Web query-based surveillance in Sweden during the influenza A(H1N1)2009 pandemic, April 2009 to February 2010. *Euro Surveillance*, 16(18), 2011. ISSN 1560-7917. PMID: 21586265.

Anette Hulth, Gustaf Rydevik, and Annika Linde. Web queries as a source for syndromic surveillance. *PLOS ONE*, 4(2):e4378, February 2009. doi: 10.1371/journal.pone.0004378. URL http://dx.doi.org/10.1371/journal.pone.0004378.

Anette Hulth, Yvonne Andersson, Kjell-Olof Hedlund, and Mikael Andersson. Eye-opening approach to norovirus surveillance. *Emerging Infectious Diseases*, 16(8):1319–1321, August 2010. ISSN 1080-6040. doi: 10.3201/eid1608.100093. URL http://www.ncbi.nlm.nih.gov/pmc/articles/PMC3298324/. PMID: 20678337 PMCID: PMC3298324.

David G. Ingram and David T. Plante. Seasonal trends in restless legs symptomatology: evidence from Internet search query data. *Sleep Medicine*, 14(12):1364–1368, December 2013. ISSN 1389-9457. doi: 10.1016/j.sleep.2013.06.016. URL http://www.sciencedirect.com/science/article/pii/S1389945713011039.

Bao Jia-xing, Lv Ben-fu, Peng Geng, and Li Na. Gonorrhea incidence forecasting research based on Baidu search data. In *Proc. Management Science and Engineering (ICMSE)*, pages 36–42. IEEE, 2013. doi: 10.1109/ICMSE.2013.6586259.

Heather A Johnson, Michael M Wagner, William R Hogan, Wendy Chapman, Robert T Olszewski, John Dowling, and Gary Barnas. Analysis of Web access logs for surveillance of influenza. *Studies in Health Technology and Informatics*, 107(2):1202–1206, 2004. ISSN 0926-9630. URL http://www.ncbi.nlm.nih.gov/pubmed/15361003. PMID: 15361003.

Min Kang, Haojie Zhong, Jianfeng He, Shannon Rutherford, and Fen Yang. Using Google Trends for influenza surveillance in South China. *PLOS ONE*, 8(1):e55205, January 2013. doi: 10.1371/journal.pone.0055205. URL http://dx.doi.org/10.1371/journal.pone.0055205.

Eui-Ki Kim, Jong Hyeon Seok, Jang Seok Oh, Hyong Woo Lee, and Kyung Hyun Kim. Use of Hangeul Twitter to track and predict human influenza infection. *PLOS ONE*, 8(7):e69305, July 2013. doi: 10.1371/journal.pone.0069305. URL http://dx.doi.org/10.1371/journal.pone.0069305.

Alex Lamb, Michael J. Paul, and Mark Dredze. Separating fact from fear: Tracking flu infections on Twitter. In *Proc. Human Language Technologies (NAACL-HLT)*, pages 789–795. North American Chapter of the Association for Computational Linguistics, 2013. URL http://www.aclweb.org/anthology/N/N13/N13-1097.pdf.

V. Lampos and N. Cristianini. Tracking the flu pandemic by monitoring the social Web. In *Proc. Cognitive Information Processing (CIP)*, pages 411–416. IEEE, June 2010. ISBN 978-1-4244-6457-9. doi: 10.1109/CIP.2010.5604088.

Vasileios Lampos and Nello Cristianini. Nowcasting events from the social Web with statistical learning. *Transactions on Intelligent Systems and Technology*, 3(4):72:1–72:22, September 2012. ISSN 2157-6904. doi: 10.1145/2337542.2337557. URL http://doi.acm.org/10.1145/2337542.2337557.

Johan Lindh, Måns Magnusson, Maria Grünewald, and Anette Hulth. Head lice surveillance on a deregulated OTC-sales market: A study using Web query data. *PLOS ONE*, 7(11):e48666, November 2012. doi: 10.1371/journal.pone.0048666. URL http://dx.doi.org/10.1371/journal.pone.0048666.

David J McIver and John S Brownstein. Wikipedia usage estimates prevalence of influenza-like illness in the united states in near real-time. *PLoS computational biology*, 10(4):e1003581, 2014.

Anna C Nagel, Ming-Hsiang Tsou, Brian H Spitzberg, Li An, J Mark Gawron, Dipak K Gupta, Jiue-An Yang, Su Han, K Michael Peddecord, Suzanne Lindsay, and Mark H Sawyer. The complex relationship of realspace events and messages in cyberspace: Case study of influenza and pertussis using Tweets. *Journal of Medical Internet Research*, 15(10), October 2013. ISSN 1439-4456. doi: 10.2196/jmir.2705. URL http://www.ncbi.nlm.nih.gov/pmc/articles/PMC3841359/. PMID: 24158773 PMCID: PMC3841359.

Seth M Noar, Kurt M Ribisl, Benjamin M Althouse, Jessica Fitts Willoughby, and John W Ayers. Using digital surveillance to examine the impact of public figure pancreatic cancer announcements on media and search query outcomes. *JNCI Monographs*, 2013(47):188–194, 2013.

Elaine O Nsoesie, David L Buckeridge, and John S Brownstein. Guess who's not coming to dinner? evaluating online restaurant reservations for disease surveillance. *Journal of medical Internet research*, 16(1), 2014.

Alex J. Ocampo, Rumi Chunara, and John S. Brownstein. Using search queries for malaria surveillance, Thailand. *Malaria Journal*, 12(1):390, November 2013. ISSN 1475-2875. doi: 10.1186/1475-2875-12-390. URL http://www.malariajournal.com/content/12/1/390/abstract. PMID: 24188069.

Michael J. Paul and Mark Dredze. You are what you Tweet: Analyzing Twitter for public health. In *Proc. Weblogs and Social Media (ICWSM)*. AAAI, 2011.

Camille Pelat, Clement Turbelin, Avner Bar-Hen, Antoine Flahault, and Alain-Jacques Valleron. More diseases tracked by using Google Trends. *Emerging Infectious Diseases*, 15(8):1327–1328, August 2009. ISSN 1080-6040. doi: 10.3201/eid1508.090299. URL http://www.ncbi.nlm.nih.gov/pmc/articles/PMC2815981/. PMID: 19751610 PMCID: PMC2815981.

Philip M. Polgreen, Yiling Chen, David M. Pennock, Forrest D. Nelson, and Robert A. Weinstein. Using Internet searches for influenza surveillance. *Clinical Infectious Diseases*, 47(11):1443–1448, December 2008. ISSN 1058-4838, 1537-6591. doi: 10.1086/593098. URL http://cid.oxfordjournals.org/content/47/11/1443. PMID: 18954267.

José Carlos Santos and Sérgio Matos. Predicting flu incidence from portuguese tweets. *Work-Conference on Bioinformatics and Biomedical Engineering (IWBBIO)*, 2013.

Samuel V Scarpino, Nedialko B Dimitrov, and Lauren Ancel Meyers. Optimizing provider recruitment for influenza surveillance networks. *PLoS computational biology*, 8(4):e1002472, 2012.

Ari Seifter, Alison Schwarzwalder, Kate Geis, and John Aucott. The utility of "Google Trends" for epidemiological research: Lyme disease as an example. *Geospatial Health*, 4(2):135–137, May 2010. ISSN 1970-7096. URL http://www.geospatialhealth.unina.it/.

Jeffrey Shaman, Alicia Karspeck, Wan Yang, James Tamerius, and Marc Lipsitch. Real-time influenza forecasts during the 2012–2013 season. *Nature Communications*, 4, December 2013. doi: 10.1038/ncomms3837. URL http://www.nature.com/ncomms/2013/131203/ncomms3837/full/ncomms3837.html.

Alessio Signorini, Alberto Maria Segre, and Philip M. Polgreen. The use of Twitter to track levels of disease activity and public concern in the U.S. during the influenza A H1N1 pandemic. *PLOS ONE*, 6(5):e19467, May 2011. doi: 10.1371/journal.pone.0019467. URL http://dx.doi.org/10.1371/journal.pone.0019467.

Brian P. Walcott, Brian V. Nahed, Kristopher T. Kahle, Navid Redjal, and Jean-Valery Coumans. Determination of geographic variance in stroke prevalence using Internet search engine analytics. *Journal of Neurosurgery*, 115(6):E19, June 2011. doi: 10.3171/2011.2.FOCUS1124. URL http://thejns.org/doi/abs/10.3171/2011.2.FOCUS1124@col.6.

Kumanan Wilson and John S. Brownstein. Early detection of disease outbreaks using the Internet. *Canadian Medical Association Journal*, 180(8):829–831, April 2009. ISSN 0820-3946, 1488-2329. doi: 10.1503/cmaj.1090215. URL http://www.cmaj.ca/content/180/8/829.

Hong-Hee Won, Woojae Myung, Gil-Young Song, Won-Hee Lee, Jong-Won Kim, Bernard J. Carroll, and Doh Kwan Kim. Predicting national suicide numbers with social media data. *PLOS ONE*, 8(4):e61809, April 2013. doi: 10.1371/journal.pone.0061809. URL http://dx.doi.org/10.1371/journal.pone.0061809.

Danqing Xu, Yiqun Liu, Min Zhang, Shaoping Ma, Anqi Cui, and Liyun Ru. Predicting epidemic tendency through search behavior analysis. In *Proc. International Joint Conference on Artificial Intelligence (IJCAI)*, pages 2361–2366. AAAI, 2011. ISBN 978-1-57735-515-1. doi: 10.5591/978-1-57735-516-8/IJCAI11-393.

Wei Xu, Zhen-Wen Han, and Jian Ma. A neural netwok [sic] based approach to detect influenza epidemics using search engine query data. In *Proc. Machine Learning and Cybernetics (ICMLC)*, pages 1408–1412. IEEE, 2010. doi: 10.1109/ICMLC.2010.5580851.

Albert C. Yang, Norden E. Huang, Chung-Kang Peng, and Shih-Jen Tsai. Do seasons have an influence on the incidence of depression? the use of an Internet search engine query data as a proxy of human affect. *PLOS ONE*, 5(10):e13728, October 2010. doi: 10.1371/journal.pone.0013728. URL http://dx.doi.org/10.1371/journal.pone.0013728.

Albert C. Yang, Shi-Jen Tsai, Norden E. Huang, and Chung-Kang Peng. Association of Internet search trends with suicide death in Taipei City, Taiwan, 2004–2009. *Journal of Affective Disorders*, 132(1–2):179–184, July 2011. ISSN 0165-0327. doi: 10.1016/j.jad.2011.01.019. URL http://www.sciencedirect.com/science/article/pii/S0165032711000528.

Qingyu Yuan, Elaine O. Nsoesie, Benfu Lv, Geng Peng, Rumi Chunara, and John S. Brownstein. Monitoring influenza epidemics in China with search query from Baidu. *PLOS ONE*, 8(5):e64323, May 2013. doi: 10.1371/journal.pone.0064323. URL http://dx.doi.org/10.1371/journal.pone.0064323.

Andrey Zheluk, Casey Quinn, Daniel Hercz, and James A Gillespie. Internet search patterns of human immunodeficiency virus and the digital divide in the Russian Federation: Infoveillance study. *Journal of Medical Internet Research*, 15(11), November 2013. ISSN 1439-4456. doi: 10.2196/jmir.2936. URL http://www.ncbi.nlm.nih.gov/pmc/articles/PMC3841350/. PMID: 24220250 PMCID: PMC3841350.

Xi-chuan Zhou and Hai-bin Shen. Notifiable infectious disease surveillance with data collected by search engine. *Journal of Zhejiang University SCIENCE C*, 11(4):241–248, April 2010. ISSN 1869-1951, 1869-196X. doi: 10.1631/jzus.C0910371. URL `http://link.springer.com/article/10.1631/jzus.C0910371`.

Xichuan Zhou, Jieping Ye, and Yujie Feng. Tuberculosis surveillance by analyzing google trends. *IEEE Transactions on Biomedical Engineering*, 58(8):2247–2254, 2011. ISSN 0018-9294. doi: 10.1109/TBME.2011.2132132.
